# Co‐building a training programme to facilitate patient, family and community partnership on research grants: A patient‐oriented research project

**DOI:** 10.1111/hex.13763

**Published:** 2023-04-20

**Authors:** Ingrid Nielssen, Sadia Ahmed, Sandra Zelinsky, Brian Dompe, Paul Fairie, Maria J. Santana

**Affiliations:** ^1^ Alberta SPOR SUPPORT Unit, Patient Engagement Team Calgary Alberta Canada; ^2^ Department of Community Health Sciences University of Calgary Calgary Alberta Canada; ^3^ Department of Pediatrics University of Calgary Calgary Alberta Canada

**Keywords:** co‐build, evaluation, grant applications, patient engagement, patient research partners, patient‐oriented research, training programme

## Abstract

**Introduction:**

Patient engagement in patient‐oriented research (POR) is described as patients collaborating as active and equal research team members (patient research partners [PRPs]) on the health research projects and activities that matter to them. The Canadian Institutes of Health Research (CIHR), Canada's federal funding agency for health research, asks that patients be included as partners early, often and at as many stages of the health research process as possible. The objective of this POR project was to co‐build an interactive, hands‐on training programme that could support PRPs in understanding the processes, logistics and roles of CIHR grant funding applications. We also conducted a patient engagement evaluation, capturing the experiences of the PRPs in co‐building the training programme.

**Methods:**

This multiphased POR study included a Working Group of seven PRPs with diverse health and health research experiences and two staff members from the Patient Engagement Team. Seven Working Group sessions were held over the 3‐month period from June to August 2021. The Working Group worked synchronously (meeting weekly online via Zoom) as well as asynchronously. A patient engagement evaluation was conducted after the conclusion of the Working Group sessions using a validated survey and semi‐structured interviews. Survey data were analysed descriptively and interview data were analysed thematically.

**Results:**

The Working Group co‐built and co‐delivered the training programme about the CIHR grant application process for PRPs and researchers in five webinars and workshops. For the evaluation of patient engagement within the Working Group, five out of seven PRPs completed the survey and four participated in interviews. From the survey, most PRPs agreed/strongly agreed to having communication and supports to engage in the Working Group. The main themes identified from the interviews were working together‐communication and supports; motivations for joining and staying; challenges to contributing; and impact of the Working Group.

**Conclusion:**

This training programme supports and builds capacity for PRPs to understand the grant application process and offers ways by which they can highlight the unique experience and contribution they can bring to each project. Our co‐build process presents an example and highlights the need for inclusive approaches, flexibility and individual thinking and application.

**Patient or Public Contribution:**

The objective of this project was to identify the aspects of the CIHR grant funding application that were elemental to having PRPs join grant funding applications and subsequently funded projects, in more active and meaningful roles, and then to co‐build a training programme that could support PRPs to do so. We used the CIHR SPOR Patient Engagement Framework, and included time and trust, in our patient engagement approaches to building a mutually respectful and reciprocal co‐learning space. Our Working Group included seven PRPs who contributed to the development of a training programme. We suggest that our patient engagement and partnership approaches, or elements of, could serve as a useful resource for co‐building more PRP‐centred learning programmes and tools going forward.

## 1 BACKGROUND

In 2015, the Canadian Institutes of Health Research (CIHR), Canada's federal funding agency for health research, introduced the Strategy for Patient‐Oriented Research (SPOR) to facilitate and fund the full spectrum of patient engagement in patient‐oriented research (POR). In POR, patients are described as individuals with lived experience of a health condition, their caregivers, family members and friends, and this can also include members of a specific affected community.^1^ Patient engagement in POR is described as patients collaborating as active and equal research team members (partners) on the health research projects and activities that matter to them.^1^ To ensure that research results reflect patient, family and community priorities and that research results can be shared and implemented in ways most appropriate to end users, CIHR SPOR asks that patients be included as research team partners early, often and at as many stages of the health research cycle processes as possible. The early stages of engaging patients in research projects include research priority setting, project design and grant funding applications.^2^

The Canadian Common CV (CCV) was launched in 2002 by Canada's Tri‐Council research agencies.^3^ The CCV is a web‐based application supporting a standardised approach for researchers to enter their academic career‐related curriculum vitae (CV) information such as education, publications and academic service.^3^ This is required on Tri‐Council grant funding applications including those submitted to CIHR. CIHR encourages patients to join health research teams on grant funding applications as Principal Co‐Applicants, Co‐Applicants, Collaborators or Knowledge Users, which requires patient research partners (PRPs) to complete specific requirements associated with those roles. Some of these requirements are acquiring a CIHR PIN #, completing the CCV, completing the Most Significant Contributions sections and writing a letter of support.^4^

However, to date, there have been very little capacity‐building opportunities to support PRPs during the grant application phase of a research study. Additionally, the process of obtaining a CCV has proven cumbersome and challenging for PRPs, specifically for those applicants without a traditional academic background.^5^ In this way, the CCV requirement acts as a barrier to engage patients meaningfully in health research projects and activities, and therefore to conduct POR. To work around this veritable patient engagement barrier, members of the Patient Engagement Team responded to the clearly articulated need to support PRPs in understanding the CIHR grant funding application process and in completing the CCV application.

The objective of this POR project was to cobuild an interactive, hands‐on training programme, with and by PRPs, a programme that could support PRPs in understanding the specific roles and processes of CIHR grant funding applications and for completing the CCV application. In this paper, we use PRPs to refer to patients, families and community members engaged in health research projects. We also conducted a patient engagement evaluation, capturing the experiences of the PRPs in cobuilding the training programme.

## 2 METHODS

This POR was a multiphased mixed‐method study. The phases included:

Phase 1: Engaged PRPs to cobuild materials, content and design format for delivery of the training programme.

Phase 2: Codeliver the training programme through webinars and workshops.

Phase 3: Evaluated patient engagement within the Working Group.

**Figure 1:**
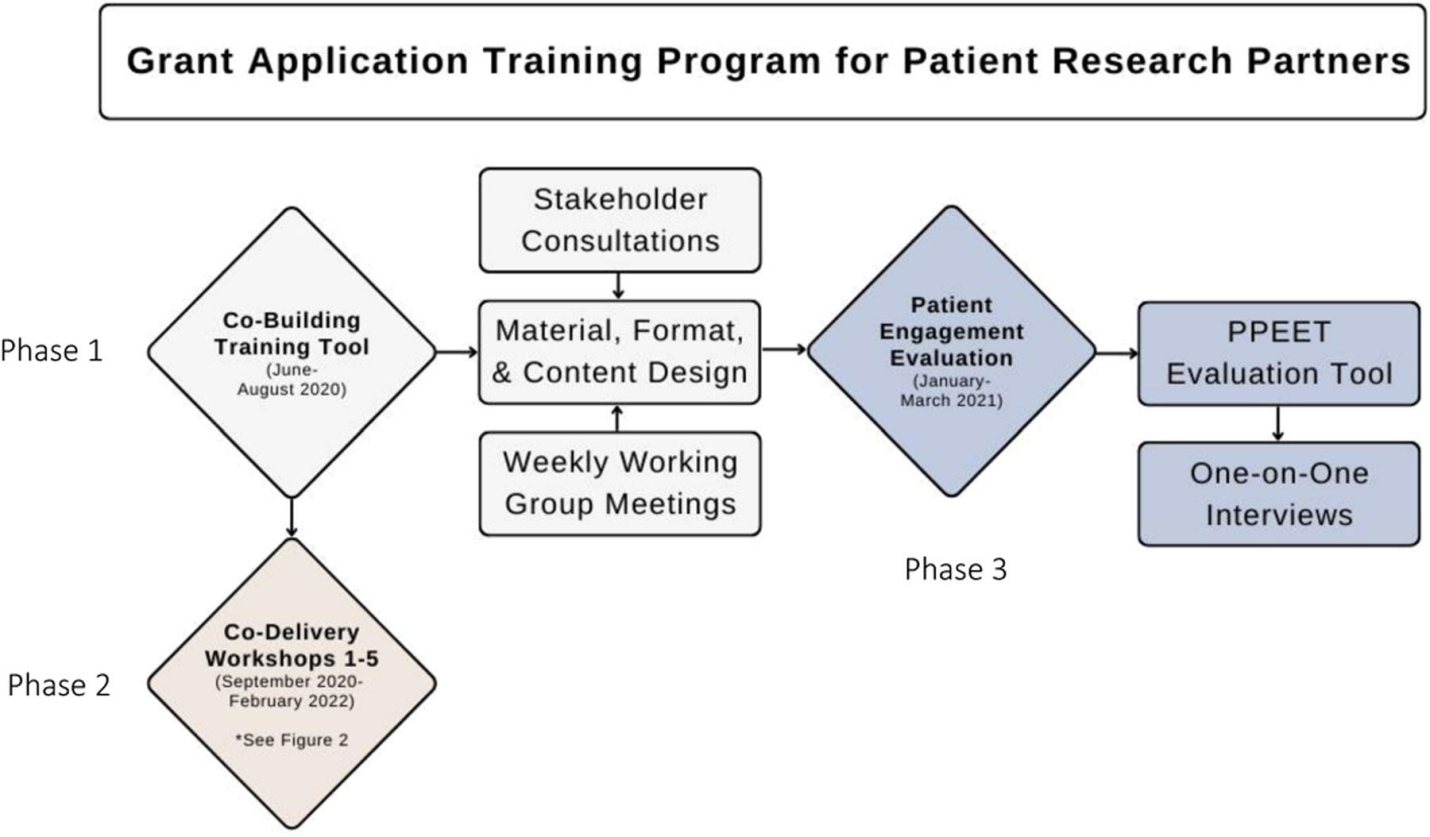
Overview of the grant application training programme process. PPEET, Patient and Public Engagement Evaluation Tool.

The study was guided by the SPOR Patient Engagement Framework, which identifies Inclusiveness, Support, Mutual Respect and Cobuild as the four guiding principles of patient engagement in POR.^1^ In this section, we describe the novel patient engagement approaches used to achieve this objective. We also share the methods used for the patient engagement evaluation of the Working Group. Figure 1 provides an overview of the project from cobuild to evaluation.

### 2.1 Phase 1: Co‐building the training programme

#### 2.1.1 Formation of the Working Group

The Patient Research Partner Lead (PRPL) and the Patient Engagement Coordinator (PEC) of the Patient Engagement Team invited PRPs who had experience working on POR projects to join a Working Group. The opportunity to join the Working Group was shared with our provincial patient and community collaborative council and through PRP networks in the province.

The Working Group included the PRPL, the PEC and seven PRPs adults (of various ages), with diverse lived experiences and conditions (cardiovascular & stroke, cancer, intensive care, inflammatory bowel disease, mental health, seniors' health, and so forth). PRPs had experience working as patient partners in research for 1–10 years. PRPs represented multiple professional and educational backgrounds, which allowed for each team member to contribute their unique skill sets and knowledge to cobuilding the training programme. Aside from the PRPL, only one other PRP of the Working Group had experience completing their CCV.

The three objectives of the Working Group were (1) to identify aspects of the CCV that are mandatory and relevant for PRPs to complete, (2) to strategize ways by which these sections could be best completed to capture and highlight the lived and other expertise that PRPs bring to research project and activities and (3) to share insights and input on additional grant submission guidance documents including writing letters of support and completing the Most Significant Contributions section.

#### 2.1.2 Working together

Because of the COVID‐19 pandemic, the project was carried out entirely online. Seven working sessions were held over the 3‐month period from June to August 2021. The Working Group worked synchronously, meeting weekly online via Zoom, as well as asynchronously, working independently to review and edit live documents via shared Google Slides and Docs working towards the common goal of cobuilding the training programme.

To support PRPs in collaborating on the cobuilding of the training programme, the Patient Engagement Team followed best practices to identify, understand and implement PRP supports. Supports included time and training to become familiar with Zoom and additional time to prepare for and establish familiarity with navigating the CCV interface. PRPs had access to a shared drive that housed all the Working Group and training materials including the slide deck presentations. PRPs were also encouraged to provide edits, comments and feedback on the training programme content and delivery via email, telephone, through Zoom meetings or via the chat option during Zoom meetings. As per the AbSPORU Patient Partner Appreciation Guidelines,^6^ compensation was offered to PRPs for their time, work and collaboration on this online project.

In preparation to introduce the CCV to the rest of the Working Group, two of the PRPs completed their CCVs early on in the process of cobuilding the training programme, which allowed them to become more familiar with the content and to capture crucial information that would later become content for the training programme.

**Figure 2:**
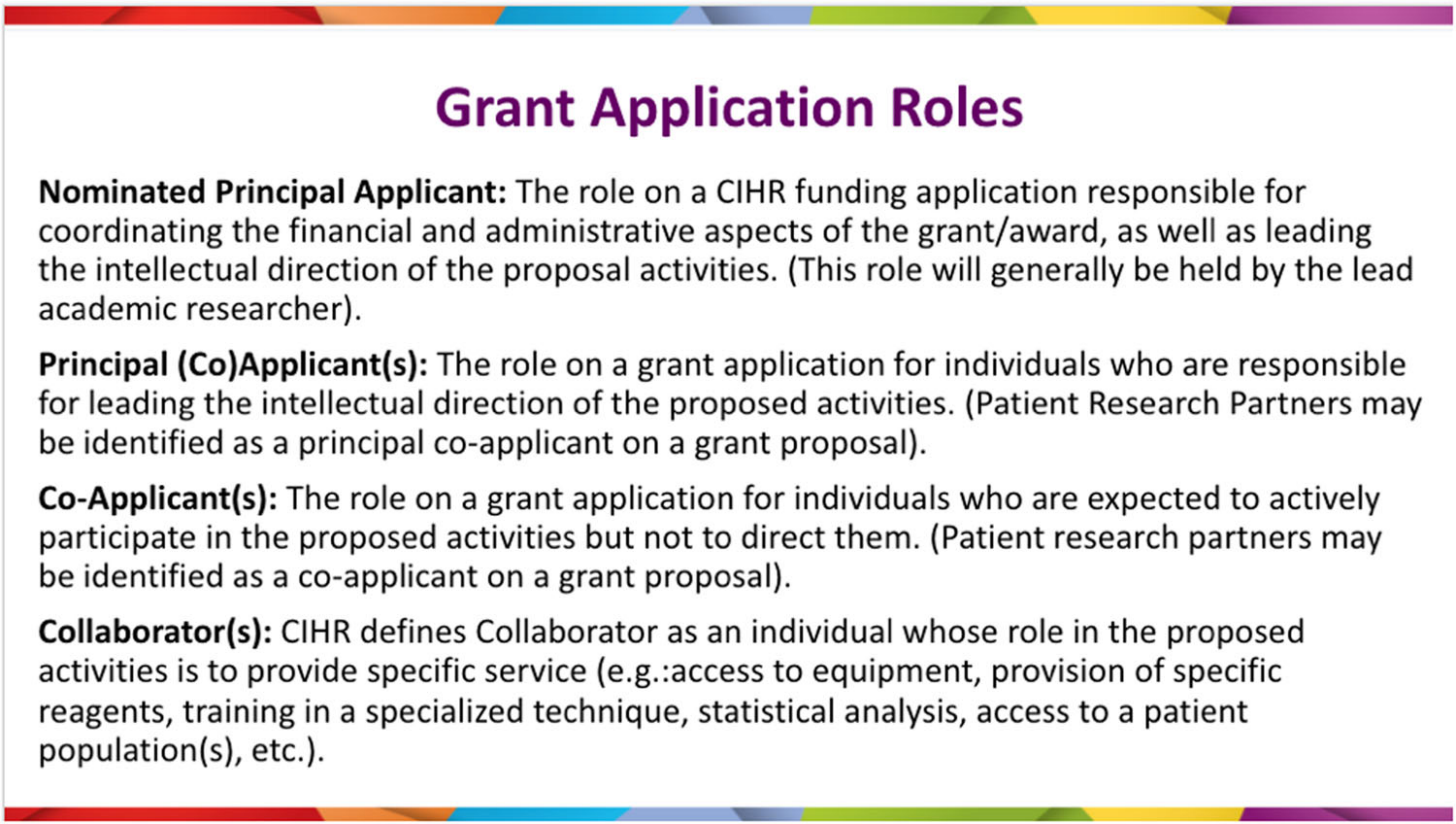
Canadian Institutes of Health Research Grant application roles.

**Figure 3:**
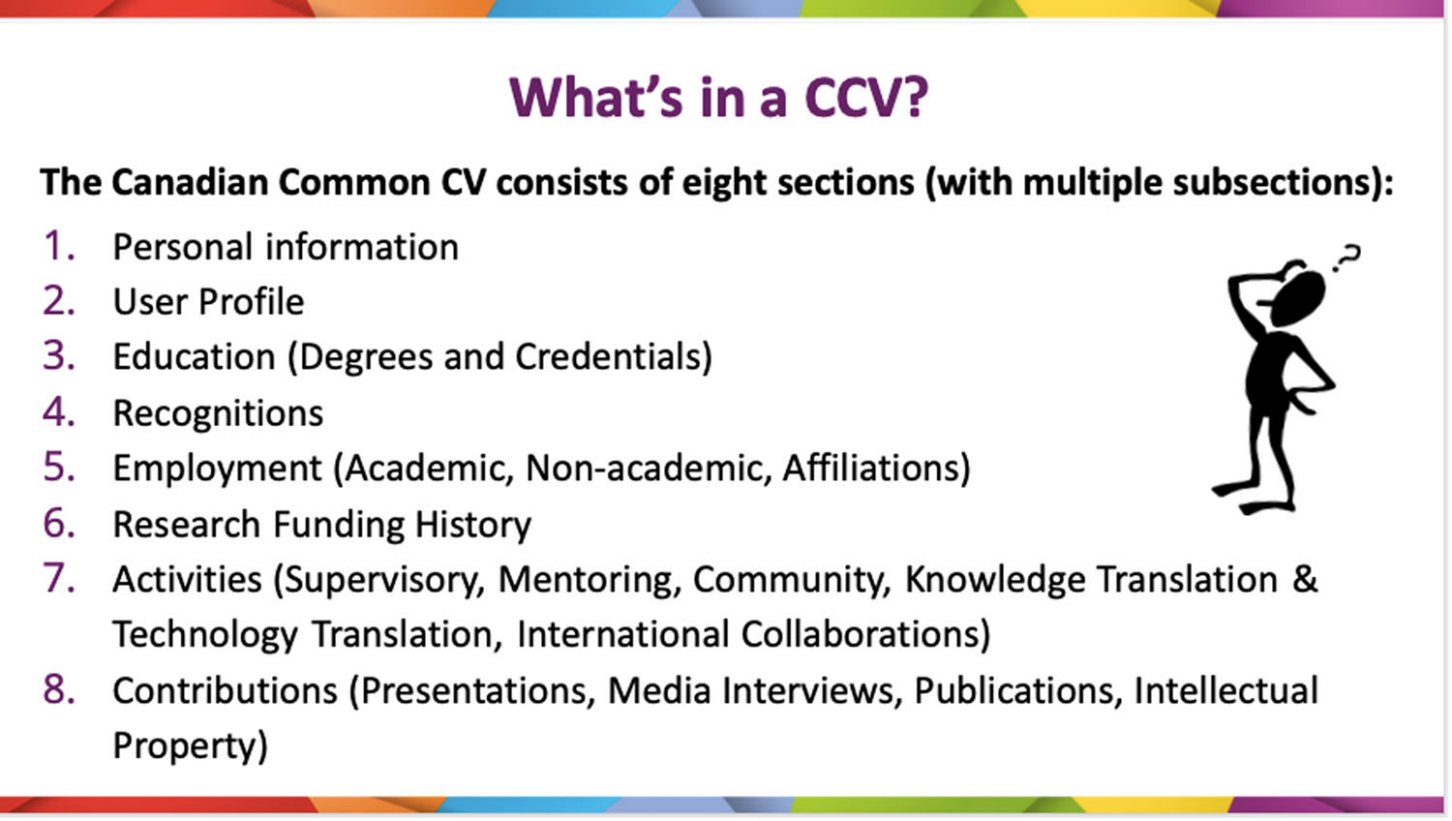
Sections of the Canadian Common CV.

The Working Group began with unpacking the CIHR grant application process, specifically identifying research team roles on CIHR grant funding applications (Figure 2) and identifying submission requirements for each of the roles. More active research team roles (i.e., that of the principal applicant or coapplicant) require the completion of the CCV application. The CCV consists of eight sections and many subsections, some mandatory and the remaining optional (Figure 3).

Aftersome Working Group members had attempted and/or completed the CCV application process, we were able to:

1. Confirm sections and subsections mandatory for PRPs to complete.
2. Generate group discussions about which additional sections could be relevant for PRPs to complete.
3. Identify opportunities and approaches to completing sections that could best highlight relevant lived experience, research experiences and additional contributions.
4. Identify additional application requirements for the various roles.

In September 2020, 3 staff members from CIHR SPOR and 11 CIHR staff members who specifically work with the CCV web‐based application were contacted to obtain feedback on the content of the training programme in a 1‐ meeting.

CIHR members were both interested in learning about the barriers that were identified by the Working Group as well as open to offering their feedback on the content that was developed thus far. The CIHR members understood the necessity for creating adaptations to sections throughout the CCV web‐based application to enable PRPs to complete the CCV. In addition, they provided feedback for considerations around privacy and the inputting of personal information on the CCV website. They also shared the newly developed Applicant Profile CV that was being piloted as an option that Knowledge Users could complete. After this consultation, some minor changes were made to the content of the training material, which included updating the content to include information about the Applicant Profile CV for Knowledge Users. The CIHR members were also invited to attend the first CCV webinar. Two CIHR members attended and subsequently sent out an email to thank and congratulate the Working Group on a job well done. At the end of the seven Phase 1 Working Group sessions, we had co‐built a training programme that included webinars and workshops ready to be delivered to other PRPs and researchers.

### 2.2 Phase 2: Delivery of the training programme

Initially, the training programme was to be delivered in two sessions: an initial 1‐h webinar that provided an overview of the CIHR grant funding application process and an orientation to the CCV interface, followed up by a 2‐h hands‐on online workshop scheduled within 2–4 weeks of the webinar.

Prerequisite work was required for the workshop aspect of the programme. To accommodate the time involved in acquiring workshop prerequisites, the PRPL sent an email a week in advance to the attendees of the workshop that detailed the steps to set up a ResearchNet account, request a CIHR PIN number (a process that can take 1–3 business days), complete the equity and diversity questionnaire and to set up a CCV account. Having these items completed in advance allowed the workshop attendees to actively work on their CCV during the workshop, allowing for necessary time and support to complete the Education, Recognitions, Employment, Research Funding History, Activities and Contributions sections.

Webinar and Workshop no. 1 was co‐presented and co‐facilitated by members of the Working Group. This was piloted to a group of seven workshop attendees who were all active PRPs, and who had not yet completed their CCV or been involved in the grant application process. A follow‐up meeting was held by the PRPL, PEP and some members of the Working Group with the workshop attendees to elicit feedback about scope, flow, content and format. The feedback was invaluable. For example, the Workshop no. 1 attendees recommended that we create a guide or template to capture CCV information such as lived health experiences, research experiences, publications, training and any other background information that would help to capture their expertise on the grant application to be included as pre‐workshop homework. These iterative improvements to the content and delivery format were made, which informed the second delivery of the training programme.

Subsequent workshops were refined and delivered by the PRPL, PEC and a PRP Working Group member, who is also a co‐author on this publication.

#### 2.2.1 Modifications made to the training programme

Members of the Working Group made note of changes and improvements that might be considered for next deliveries or unique audiences. For example, based on feedback from the workshop attendees who indicated that they would prefer to complete everything in one workshop, it was decided to deliver the programme in a 2.5‐h workshop (rather than a 1‐h webinar, followed up by a 2‐h workshop). Participant recommendations to send out a guide or template document as pre‐workshop homework where participants could consolidate their relevant and research experience for an easy copy/paste at the workshop further supported this decision.

The 2.5 h workshop format was then delivered to a National SPOR Research Network (Workshop no. 3), a Provincial SPOR SUPPORT Unit (Workshop no. 4) and a rare disease research network to support caregiver PRPs on a grant funding application (Workshop no. 5). These workshops were not formally evaluated; however, the PRPL and PEC did ask for feedback and comments during and after the workshop delivery and paid regular attention to email notes that identified gaps or processes for registration, workshop preparation and follow‐up that could be improved going forward.

### 2.3 Phase 3—Evaluation of the engagement of the working group

To evaluate the experiences of PRPs in the Working Group, a qualitative researcher (MSc trained) from the Patient Engagement Team (who was not a member of the Working Group) administered the Patient and Public Engagement Evaluation Tool (PPEET) via a secure online Qualtrics anonymous survey to the PRP members of the Working Group (*n* = 7), at the conclusion of the Working Group sessions.^7^ The researcher also invited PRPs of the Working Group to participate in semi‐structured one‐on‐one interviews following the survey distribution. Informed consent was obtained from all the PRPs included in the evaluation. The Institutional Health Research Ethics Board (CHREB) approved the evaluation study (REB20‐1822).

The PPEET is a validated tool co‐developed by Canadian researchers and public and patient engagement practitioners.^7^ PPEET questions reflect the core principles of high‐quality patient engagement. Therefore, module B of the PPEET, which assesses ongoing/long‐term engagement, was administered to the PRPs.

Survey questions aimed to understand the experience and impact of engagement in the following key areas: communication and supports for participation; sharing your views and perspectives; and impacts and influence of the engagement initiative. The survey consisted of 22 questions/statements rated using a five‐point Likert scale from *strongly agree* to *strongly disagree*, and open text boxes for further comments. An additional open‐text question was added to the survey on whether the PRPs considered leaving the group at any point, and if so, why.

Afterwards, PRPs were invited to share their experiences further in an online one‐on‐one interview. The semi‐structured interview guide was co‐developed with PRPs in Alberta. Interview guide questions complemented the questions from the PPEET survey, with a focus on members' experiences working with each other, how they felt supported, barriers to engaging in the work and their motivations for joining and staying with the Working Group. PRPs were offered a $20 gift card in appreciation for their contribution to the survey and interview.

Survey responses were collected and stored on the online Qualtrics platform.^8^ Descriptive statistics (frequencies) were summarised for the quantitative responses and qualitative responses were summarised per section. Interviews were transcribed, anonymized and thematically analysed by two qualitative researchers using a mix of deductive and inductive coding strategies. The domains of the PPEET tool and CIHR Patient Engagement Framework informed the codes identified.^1^ We followed the steps of thematic analysis outlined by Braun and Clarke.^9^ Findings from the surveys and interviews were compared and summarised in descriptive tables. We used strategies to increase credibility, such as member checking (asking participants to clarify responses during the interviews). Member checking ensured that participants were given the opportunity to confirm their responses and support the accuracy of interpretation in data analysis. We also used peer debriefing between team members to discuss the feedback provided by PRPs of the Working Group and the main themes identified. Team member discussions about identified themes and supporting quotes helped to confirm whether the findings made sense and whether the themes accurately captured the associated quotes.

## 3 RESULTS

Over the 2‐year period, the Working Group co‐built a PRP‐informed training programme about the grant funding application process to address a veritable institutional barrier to the authentic, meaningful inclusion of PRPs in essential early stages of research project priority setting, design and grant funding applications. All webinar and workshops were co‐delivered by members of the Working Group (PRPL, PEC and PRP co‐author of this paper). Format for delivery, including length of time, of the training programme was adjusted slightly to recognise unique audiences such as patient groups or research teams. The content of the training programme included an overview of the grant application process and roles, components of the CCV and Applicant Profile CV, guidance about how to complete the sections of the CCV, how to complete the Most Significant Contributions section and tips for writing a letter of support.

**Figure 4:**
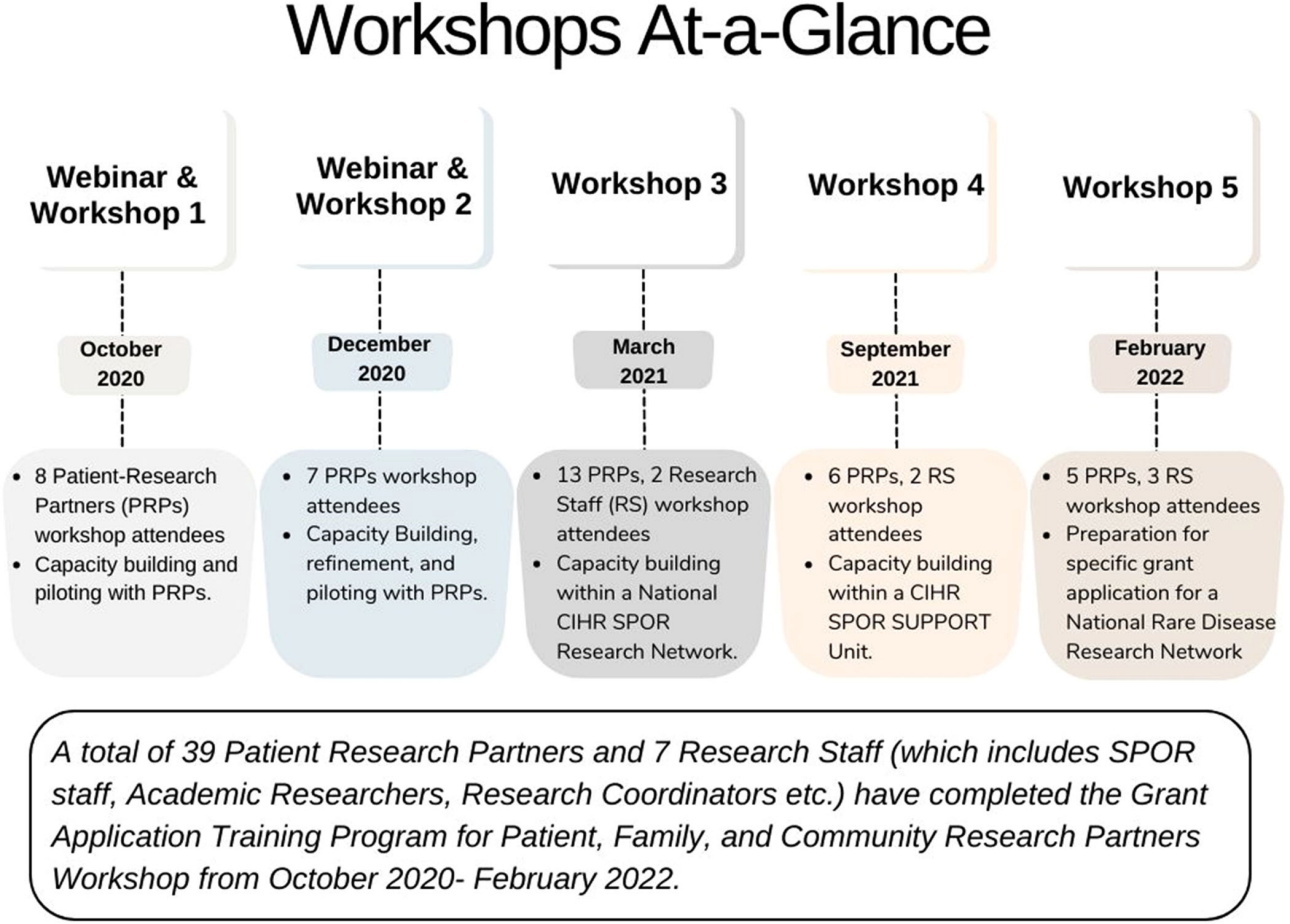
Overview of the workshops co‐delivered by members of the Working Group.

A total of 39 PRPs and 7 research staff have completed the training programme from October 2020 to February 2022. Figure 4 provides an overview of the workshops that have been delivered. CIHR continues to be responsive to questions that have arisen over the 2 years that the workshops have been delivered.

### 3.1 Evaluation of the working group

Of the seven PRPs of the Working Group, five completed the evaluation survey and four participated in one‐on‐one interviews. Evaluation results from the PPEET survey are summarised in Table 1, and themes and subthemes identified from the interviews are summarised in Table 2. The findings from both the survey and interviews are also described and compared below.

**Table 1:** Results from Surveying Patient Research Partners on Engagement via PPEET (*n* = 5 PRP respondents out of 7 PRP Working Group members).

	Strongly agree *n* (%)	Agree *n* (%)	Neither agree nor disagree *n* (%)	Disagree *n* (%)	Strongly disagree *n* (%)
I have a clear understanding of the purpose of the Canadian Common CV webinar and workshop.	3 (60)	2 (40)	–	–	–
The supports I need to participate in the Working Group are available (e.g., travel, childcare, technology and internet support).	1 (20)	3 (60)	1 (20)	–	–
I have enough information to be able to carry out my role.	1 (20)	4 (80)	–	–	–
I am able to express my views freely.	3 (60)	2 (40)	–	–	–
I feel that my views are heard.	2 (40)	3 (60)	–	–	–
A wide range of views on discussion topics is shared.	2 (40)	3 (60)	–	–	–
The individuals participating in the Working Group represent a broad range of perspectives.	1 (20)	2 (40)	2 (40)	–	–
This Working Group is achieving its stated objectives.	1 (20)	3 (60)	1 (20)	–	–
I am confident that the Canadian Institutes of Health Research (CIHR) takes the feedback provided by the Working Group into consideration.	–	2 (40)	3 (60)	–	–
I think that the work of the Working Group makes a difference to the work of the CIHR.	3 (60)	–	2 (40)	–	–
As a result of my participation in the Working Group, I am better informed about the Canadian Common CV process for patient partners.	2 (40)	1 (20)	2 (40)	–	–
Overall, I am satisfied with this engagement initiative.	2 (40)	3 (60)	–	–	–
This engagement was a good use of my time.	1 (20)	4 (80)	–	–	–

Abbreviations: PPEET, Patient and Public Engagement Evaluation Tool; PRP, patient research partner.

**Table 2:** Themes and quotes on Patient Research Partner Experiences within the Working Group.

Themes and subthemes	Supporting quotes
Communication and supports	‘It was quite well with this kind of a progressive engagement … the Working Group that got together actually developed the …terms of reference to go forward. So it wasn't something that was just kind of laid on us. It was something we all talked about. Developing together which was a good process’. (Patient Research Partner)
	‘I appreciated the acknowledgement and recognition of my (& my colleagues) investment of time, energy & expertise in terms of having the option to be paid monetarily for this’. (Patient Research Partner)
	‘the conversations we had in creating the webinar and workshop… everyone was heard and respected’. (Patient Research Partner)
Motivations for joining and staying Engaged leadership Learning opportunity Inclusivity	‘she [Patient Research Partner Lead] comes to her role with an ease and has really good abilities to include everybody’. (Patient Research Partner)
	‘we were all engaged in the process of making decisions…so what we talked about doing, we did’. (Patient Research Partner)
	‘I have learned a lot more about the CCV, the CIHR and…I had a lot opportunities to learn a lot more about the research projects that are going on’. (Patient Research Partner)
	‘looking at the CCV and potentially making it more about, you know, capturing the lived experience of the public that are looking at wanting to get involved in research. I was intrigued on…how we could do that’. (Patient Research Partner)
Challenges to contributing	‘I wasn't able to contribute and participate as getting towards the end where they were bringing in people to help them assist in filling out the online information. That part I couldn't contribute to because I couldn't attend all the meetings’. (Patient Research Partner)
	‘Everyone there already had experience with the CCV. And for myself in the beginning, I was a little reluctant and more likely to ask questions than contribute or add to the meeting. I've never been too shy to ask questions and share my opinions. As I became more comfortable with the group, I started contributing and adding to the team’. (Patient Research Partner)
	‘worked together with some people getting stuck and the presenter trying to move them forward. And it's a delicate situation, right? Because you want to hear them out, but then you have to say, well, hang on, we've gotta get to the next step and some people have very strong personalities and they knew that if they continued to just talk and talk and talk. They would get more time to talk and others would sit back and go. Well, is this gonna get us anywhere?’ (Patient Research Partner)
Impact of the Working Group	‘I think it's probably increased the awareness. Um, certainly within [provincial] SPOR. But also with CIHR that there's an opportunity to you know, look at this thing called the CCV, and make it more acceptable. Make it more comfortable for the member of the public with the lived experience to complete. So I think it's increased the awareness’. (Patient Research Partner)
	‘The workshops we are doing in March are all for primarily researchers, so I think that it's really important to have someone like myself who is not an academic. No medical background, but actually the type of patient…patient partner that they would be dealing with in the research projects. I think that's been helpful, I also have good feedback from the people I presented to and that they really thought it was quite valuable’. (Patient Research Partner)

Abbreviations: CCV, Canadian Common CV; CIHR, Canadian Institutes of Health Research.

### 3.2 Working together—Communication and supports

Four out of five PRP survey respondents agreed/strongly agreed that they have a clear understanding of the purpose of the CCV webinar and workshop, have supports available to contribute to the Working Group, have enough information to be able to carry out their role, are able to express their views freely and feel that their views are heard.

During the interviews, PRPs of the Working Group offered additional insights into the communication and supports provided for working together. PRPs of the Working Group mentioned co‐building the workshop and webinar to be a positive experience, as they were involved from the very beginning. Members of the group included the following as key strengths of the Working Group: commitment to the project, open communication and common goals and understanding among the members.

One PRP commented ‘the conversations we had in creating the webinar and workshop … everyone was heard and respected’.

PRPs also highlighted some challenges, which included time management of the group (some members bringing up tangential topics) and the CCV being a new topic to some who were not familiar with the process. Scheduling of meetings was also mentioned by one member to be a barrier for them, as they worked full time and were unable to commit fully.

### 3.3 Motivations for joining and staying

PRPs identified drivers of engagement within the text responses of the survey as well as during the interviews. Drivers of engagement from the perspectives of the PRPs were the engaged leadership of the PRPL and PEC, feeling of inclusivity within the group, responsiveness of the team, mutual respect/working together and providing a learning opportunity.

One PRP Working Group member mentioned how they heard about the Working Group from CIHR after expressing their concerns about the grant application process being difficult for PRPs. Another member mentioned how they viewed joining the group as a learning opportunity:

‘I thought I wasn't sure how I got involved in this because it was way over my head. I have no international affiliations; I have no publication… but as it turns out, was [an] interesting experience for me to go through finding out that now you can customise it to be reflective of your personal experiences’.

Another PRP mentioned wanting to join the Working Group because they were frustrated by researchers reaching out to them at the last minute to be a part of grant applications, but providing no support to fulfil the requirements.

‘I was frustrated by …I'd be asked and then at the 11th hour you know the grants going in two days and …I get an email saying oh, we need you to do a CCV and I'm thinking well, what's a CCV and…it's not that easy, right? It's complicated…and the content that's asked for is very applicable obviously for researchers you know they ask for your academic credentials and all the history of your research engagement. But the vast majority of it … has very little applicability to the public’.

### 3.4 Impact of the Working Group

On the topic of impact of the Working Group, four out of five PRP respondents in the survey agreed/strongly agreed that the group is achieving its stated objectives. There were mixed responses from PRPs of the Working Group on whether they were confident that CIHR took the feedback provided by the Working Group into consideration (three neutral, two agree), as well as whether the work of the Working Group makes a difference to the work of CIHR (two neutral, three strongly agree). One person answered neutral to some of the statements addressing the impact of their work as they explained that they have no experience with CIHR or understanding of the level of impact of the Working Group.

One PRP commented, ‘I'm very proud of the leadership of this Working Group and feel quite privileged to be involved… at the very least, I believe the work of our group has created some awareness and interest in members of the public with a lived healthcare experience in getting their CCV created to be involved in research. I sense our work may also have increased the awareness in CIHR that making the CCV tailored to a format to members of the public with a lived healthcare experiences may be very significant in engaging more members of the public in research’.

The survey results highlight positive PRP experiences with communication and supports for working together, views on the impacts and influence of the Working Group and overall satisfaction with this engagement initiative. From the interview findings, we gain a better understanding of what aspects of the engagement initiative the PRPs valued, for instance, the specific supports that they received, their motivations for joining, the challenges to contributing to this work and further understanding on the impact of the Working Group. Both the survey and qualitative findings complement the evaluation of patient engagement within the Working Group.

## 4 DISCUSSION

The results of this project suggest that codesigned solutions in the health research ecosystem do not need to be limited to traditional research projects or healthcare service design innovations. Co‐building can be integral to success in identifying and addressing systematic and structural barriers to early patient engagement on health research projects. Including PRPs early in POR priority‐setting processes and project design is elemental to producing research evidence that translates into more patient‐centred healthcare planning, policy and practice.^1^ Identifying PRPs on grant funding applications is an essential first step to greater scope of inclusion of PRP members on health research teams. However, for this inclusion to be authentic, it is essential that PRPs have equitable access to the information and tools necessary to collaborate at important early phases including project design and grant application. To collaborate in ways that are nontokenistic and meaningful to them, PRPs must have an established understanding of the grant funding application processes, applicant roles and have the training necessary to identify and work through the more academically oriented requirements to join health research teams applying for funding and carrying out subsequently funded projects.

The purpose of the project was twofold. Guided by the CIHR SPOR Patient Engagement Framework, the PRPL and the PEC of the Patient Engagement Team worked with PRPs through a series of seven Working Group meetings to establish understanding of the CIHR grant funding application processes that were relevant to PRPs. This included identifying the opportunities and requirements associated with the various research team processes and roles associated with grant funding applications and then to identify the specific barriers that PRPs might experience in completing the CCV process. The Working Group then developed a more PRP‐friendly training programme to support more universal completion of the CCV process, ways that highlight the unique and expansive lived experience and other expertise that each PRP can bring to health research projects.

## 5 WHAT WE KNOW

PRPs are increasingly being included in health research activities beyond health research projects. This includes involvement in reviews,^10^ as co‐authors on publications^11^, ^12^ and in the co‐building of educational tools.^13^ Training for public and patient involvement (patient engagement) should be co‐produced with patients.^13^ The four CIHR SPOR PE Framework guiding principles aligned with the objective and focus of the Working Group and provided the blueprint for working together.^1^ These are detailed and their alignment is explained below.

### 5.1 Inclusiveness

Diversity in research teams and research projects can lead to more universally appropriate and applicable research results.^14^, ^15^, ^16^ There can also be inherent challenges in working together when team members reflect multiple perspectives, identities, skill sets and approaches to learning. The aim of inclusiveness is to create welcoming spaces for all individuals of diverse motivations, backgrounds and perspectives to work together in ways that are respectful and meaningful on the health research projects that matter to them. However, welcoming invitations are not enough. Creating research spaces that are void of power differentials and that instead work to support and empower a POR lens and experiential knowledge contributions are as essential as the trust necessary to encourage generative dialogue and to sustain respectful, collaborative and effective research teams.^1^, ^15^, ^17^ Black et al.,^15^ in their study, found that a research environment that was inclusive of patient partner perspectives and provided a welcoming atmosphere was important in fostering meaningful engagement. Santana et al.^16^ recommend that research teams need to embrace wide‐ranging diversity of patient partners, and supports should be offered for patient partners to collaborate comfortably and equitably. PRPs in our Working Group and participants in the workshop represented multiple educational, professional and health research backgrounds as well as varying lived experiences with health conditions and health care. They brought these multiple perspectives to the Working Group and were encouraged to share their ideas in this collaborative work.

### 5.2 Support

Support is defined as adequate support and flexibility for patient partners to ensure that they can contribute to health research projects and activities in equitable and meaningful ways.^1^, ^16^ Support refers to the creation of a safe environment that promotes equitable and honest interactions, cultural responsiveness, appropriate training, education and financial compensation for patient partner contributions. Identifying supports requires active listening to understand patient partner motivations for joining health research projects and the supports that can best foster positive engagement and colearning experience. McCarron et al.^10^, ^18^ describe providing training, technology supports, team meetings and compensation for their patient partners to engage in conducting a scoping review. Our POR project was conducted in the early phases of the COVID pandemic; therefore, extra steps were taken to ensure that PRPs were familiar with all the features of Zoom and there were constant check‐ins during meetings and workshops to support working together in virtual spaces. Compensation was offered to partners for their co‐build and co‐delivery work. The project was conducted entirely online, so there were no direct expenses to engagement.

### 5.3 Mutual respect

Relationships built on time and trust are essential to working together.^1^, ^18^ Mutual respect is when researchers, practitioners and patients acknowledge each other's expertise and experiential knowledge.^1^ Mutual respect and acknowledging patient partner expertise have been highlighted as important in many patient engagement frameworks.^19^, ^20^ In our POR project, clarity about roles and expectations was established early and the PRPL and PEP regularly checked in with PRPs of the Working Group to ensure that expectations and needs were being met. Special consideration was taken to respect the confidentiality of each member's CCV information, their lived health and health research experience and progress in completion of the CCV.

### 5.4 Co‐build

Co‐build is defined by CIHR SPOR as patients, researchers and practitioners working together from the beginning to identify problems, gaps in knowledge, to set priorities for research and to work together to produce and implement solutions.^1^ Bell et al.^13^ describe co‐building a Foundations of POR curriculum with patient partners in their study. They describe partners feeling engaged through a feedback loop across the development team and also working independently on their own content pieces.^13^ In our POR project, our Working Group worked together to establish their ‘working together’ guidelines and to identify the process steps to meet the project objectives. Working Group meetings were scheduled at a time convenient for most members to assure equitable access to collaboration. Time was taken at the beginning and end of each meeting for member check‐ins and open discussion. Members also had the opportunity to provide feedback via email if they were not able to attend meetings. Decisions were by consensus and all members were informed and gave approval at each step.

## 6 IMPLICATIONS FOR PATIENT ENGAGEMENT PRACTICE

Guided by the CIHR SPOR Patient Engagement Framework, we undertook a project to co‐build a training programme for equitable and meaningful partnership at the early stages of POR projects and to assure more meaningful patient partner roles, should projects become funded. This POR project was developed based on PRP experiences of attempting to collaborate on grant funding applications and the barriers experienced by these PRPs as they tried to do so. This format could have applicability to co‐developing PRP approaches to resolving institutional barriers to patient engagement in other contexts and more globally. Additionally, researchers hoping to partner with PRPs in health research projects could learn from the strategies that we have outlined in this paper.

## 7 STRENGTHS AND LIMITATIONS

A strength of this work is the POR design that engages patients as active partners in the co‐building of a training programme that ultimately became useful for PRPs and even clinician–researchers. We aimed to adhere to the CIHR guiding principles for patient engagement (inclusiveness, support, mutual respect, co‐build). A strength of this work is evaluating our patient engagement process by conducting surveys and interviews with our PRPs of the Working Group. The evaluation of the partnerships showed that our process was seen as meaningful to PRPs and that they felt their contribution and collaboration contributed to a more PRP‐friendly and usable approach to a barrier to engagement. Our paper demonstrates approaches that can identify and inform more patient partner‐centred cobuild projects and activities; how to work and learn together; and processes to break down research process to identify PRP aspects and then collaboratively identify barriers and how to overcome them.

The national CIHR CCV is a bilingual application with interfaces in both English and French. Our training programme was developed with English‐speaking patients only and using only the English interface. A future direction of this work would be to translate the programme for French‐speaking individuals. Another limitation of this work is that not all PRP members of the Working Group participated in the patient engagement evaluation following their engagement. We did not ask PRPs their reasons for not participating in the evaluation as the surveys were anonymous, and may have missed some valuable perspectives on engagement. Additionally, our PRP Working Group members were limited to PRPs based in Alberta. While we have evaluated the process of working together and health research partnerships within the Working Group, evaluation of the workshops from participants' perspectives has not occurred.

## 8 CONCLUSION

A POR patient engagement approach can be effective to including PRPs in projects aimed at addressing barriers to engagement that impact them. Being innovative and responsive to PRPs voice and priority can result in the novel design of a patient‐oriented training programme universal to multiple audiences. Working together, providing time, flexibility and appropriate supports for building meaningful relationships are essential to iterative cobuild work. This has resulted in an ‘idea to actioned’ hands‐on training programme for PRPs to squash a barrier to equitable research team partnership.

## AUTHOR CONTRIBUTIONS

*Contributions to conception and design*e: All authors. *Cobuilding the training program*: Ingrid Nielssen, Sandra Zelinsky, Brian Dompe, Maria J. Santana. *Conducting the evaluation, analysis, and interpretation of the data*: Sadia Ahmed, with feedback from all authors. *Drafting the manuscript*: Ingrid Nielssen, Sadia Ahmed, Sandra Zelinsky. *Revising it critically for important intellectual content*: Ingrid Nielssen, Sadia Ahmed, Sandra Zelinsky, Paul Fairie, Brian Dompe, Maria J. Santana. *Final approval of the version to be submitted*: All authors.

## CONFLICT OF INTEREST STATEMENT

The authors declare no conflict of interest.

## ETHICS STATEMENT

The evaluation study was approved by the University of Calgary Conjoint Health Research Ethics Board (CHREB) (REB20‐1822). All patient research partners gave informed consent before participating in the evaluation.

## Data Availability

Data are available on request to the authors.
